# Activation-Induced Cytidine Deaminase Alters the Subcellular Localization of Tet Family Proteins

**DOI:** 10.1371/journal.pone.0045031

**Published:** 2012-09-17

**Authors:** Yuko Arioka, Akira Watanabe, Kuniaki Saito, Yasuhiro Yamada

**Affiliations:** 1 Department of Reprogramming Science, Center for iPS Cell Research and Application (CiRA), Kyoto University, Kyoto, Japan; 2 Human Health Sciences, Graduate School of Medicine and Faculty of Medicine, Kyoto University, Kyoto, Japan; 3 Institute for Integrated Cell-Material Sciences (WPI-iCeMS), Kyoto University, Kyoto, Japan; Bellvitge Biomedical Research Institute (IDIBELL), Spain

## Abstract

Activation-induced cytidine deminase (Aid), a unique enzyme that deaminates cytosine in DNA, shuttles between the nucleus and the cytoplasm. A recent study proposed a novel function of Aid in active DNA demethylation via deamination of 5-hydroxymethylcytosine, which is converted from 5-methylcytosine by the Ten-eleven translocation (Tet) family of enzymes. In this study, we examined the effect of simultaneous expression of Aid and Tet family proteins on the subcellular localization of each protein. We found that overexpressed Aid is mainly localized in the cytoplasm, whereas Tet1 and Tet2 are localized in the nucleus, and Tet3 is localized in both the cytoplasm and the nucleus. However, nuclear Tet proteins were gradually translocated to the cytoplasm when co-expressed with Aid. We also show that Aid-mediated translocation of Tet proteins is associated with Aid shuttling. Here we propose a possible role for Aid as a regulator of the subcellular localization of Tet family proteins.

## Introduction

DNA methylation is a stable epigenetic feature that is involved in gene silencing and the maintenance of long-lasting cell memories [1]. Dynamic regulation of the DNA methylation pattern is crucial for mammalian development, as well as differentiation and reprogramming [2], [3]. In particular, the active loss of 5-methylcytosine (5mC) independent of cell division is considered to be a major initial event in the epigenetic reprogramming of early mammalian embryos [4]. It has been demonstrated that the loss of 5mC at the paternal pronucleus of a zygote is linked to the accumulation of 5-hydroxymethylcytosine (5hmC) [5]–[7]. The 5hmC is converted from 5mC by the ten-eleven translocation (Tet) family of proteins [8], and therefore 5hmC is considered to be an intermediate formed during the active DNA demethylation process in early embryos.

A recent study proposed a novel model for the removal of 5hmC, wherein activation-induced cytidine deaminase (Aid) induces the deamination of 5hmC, which is followed by base excision repair (BER), resulting in the conversion of 5hmC into unmethylated cytosine [9]. Based on this model for active DNA demethylation, coordinated actions of both the production and removal of 5hmC may regulate the conversion of 5mC into unmethylated cytosine. However, little is known how these proteins involved in the production and removal of 5hmC affect each other.

Aid is a well-known enzyme that converts cytosine into uracil in single-stranded DNA, causing somatic hypermutation and class switch recombination [10], [11]. Aid is mainly localized in the cytoplasm under steady state conditions, but has the ability to shuttle between the nucleus and the cytoplasm [12], [13]. Previous studies suggested that the changeable localization of Aid, which is mediated by its shuttling, plays a role in controlling its activity as a DNA modifier [14], [15]. Considering that 5hmC is localized at the nucleus, the shuttling of Aid may also contribute to the modulation of 5hmC removal. In addition, Tet family proteins show translocation into the nucleus from the cytoplasm during the early developmental stage, when the rapid generation of 5hmC is observed [16]. Therefore, it is possible that distinct subcellular localization of the Tet family and Aid controls the production and removal of 5hmC, leading to the regulation of active DNA demethylation. In the present study, we examined the relationship between the Tet family and Aid from the view of their subcellular localization. We herein demonstrate that Aid has an effect on the subcellular localization of the Tet family, and that this is associated with Aid shuttling.

## Materials and Methods

### DNA constructs

Mouse Tet1 (GU079948, DDBJ), Tet2 (GU079949, DDBJ), Aid (NM_009645.2, NCBI), Apobec1 (NM_031159.3, NCBI) and Apobec2 (NM_009694.3, NCBI) were cloned from mouse embryonic stem cells, and Tet3 (NM_183138.2, NCBI) was obtained from mouse embryonic fibroblasts by PCR amplification with KOD plus Neo (TOYOBO) using primers as described in Table S1. The Tet family fragments were subcloned into pcDNA4HisMax (Life Technologies), and the Aid, Apobec1 and Apobec2 fragments were subcloned into pcDNA4MycHis (Life Technologies). Plasmids encoding the Xpress-tagged catalytic domain (CD) of Tet1 (1367–2039 amino acids: aa), Tet2 (1044–1921aa) and Tet3 (697–1668aa) were generated by subcloning of the DNA fragments into BamHI and NotI sites for Tet1, EcoRI and XhoI sites for Tet2 or EcoRI and NotI sites for Tet3 of pcDNA4HisMax. Plasmids encoding Xpress-tagged mutants deficient in the catalytic domain (ΔCD) of Tet1 (1–1366aa), Tet2 (1–1043aa) and Tet3 (1–696aa) were also generated. The Aid mutants, AidΔNES (1–187aa) and AidΔN26 (27–198aa), were subcloned into BamHI/XhoI-digested pcDNA4MycHis. We used the KOD plus mutagenesis kit (TOYOBO) to generate a point mutant for Aid (F193A) and mutants for the Tet family catalytic domain, which include Tet1CDm (D1652Y, D1654A), Tet2CDm (H1304Y, D1306A) and Tet3CDm (H950Y, D952A).

### Cell culture and cDNA transfection

Human embryonic kidney cells (HEK293FT) (Invitrogen) and human colon cancer cells (DLD-1) (American Type Culture Collection) were maintained in Dulbecco's modified Eagle's medium with 10% heat-inactivated fetal bovine serum. Both of them were transiently transfected with plasmid DNA by FuGENE6 (Promega) according to the manufacturer's instructions, followed by immunofluorescence or co-immunoprecipitaion 48 h post-transfection, unless otherwise noted.

### Immunofluorescence

The cells were fixed and permeabilized with cold 100% methanol for 10 min on ice. For 5hmC staining, permeabilized cells were treated with 4 N HCl for 10 min, followed by 1.5 M Tris-HCl (pH 8.8) treatment for 10 min, before being blocked with 1% BSA. The cells were incubated with primary antibodies; anti-Xpress mouse monoclonal antibody (mAb) (Life Technologies), anti-Myc rabbit polyclonal antibody (MBL), anti-5hmC rabbit polyclonal antibody (Active Motif) or anti-Aicda rabbit polyclonal antibody (Abcam) overnight at 4°C, followed by Alexa Fluor-conjugated secondary antibodies (Life Technologies) for 1 h, and DAPI staining for 5 min at room temperature. After washing with PBS containing with 0.05% Tween 20, the samples were mounted by using the Prolong Gold Antifade Reagent (Life Technologies), followed by curing on a flat surface in the dark overnight at 4°C. For four color staining, a Zenon Alexa Fluor labeling kit (Life Technologies) was used. The images were captured by a confocal laser microscope (OLYMPUS, FV1000). To score the subcellular localization in DLD-1 cells, we counted all of the fluorescence positive cells on 4-well chamber dishes (BD). When using HEK293FT cells, we counted cells in randomly acquired fields on the 4-well chamber dishes. In the case of co-transfection, co-expressed cells were counted and scored according to the Tet localization. Scoring of the subcellular localization was performed as indicated in Fig. S1.

### Immunoprecipitation and immunoblotting

Transfected HEK293FT cells were lysed in EBC buffer (50 mM Tris-HCl pH 8.0, 120 mM NaCl and 0.5% NP40 for detecting Xpress-tagged protein, or 1.0% NP40 for detecting Myc-tagged protein) with a protease inhibitor cocktail (SIGMA). The cell lysates were incubated for 3 h at 4°C with Dynabeads M280 sheep anti-mouse IgG (VERITAS) which had been pre-treated with an anti-Xpress mAb or an anti-Myc mAb (Enzo life science) for 1 h. After washing the immunoprecipitates four times with EBC buffer, the beads were boiled with Laemmli SDS-sample buffer. This supernatant was separated by SDS-PAGE and transferred to a PVDF membrane (Millipore). For immunoblotting of the Xpress-tagged protein, after the membrane were blocked with 2% nonfat dry milk in PBS containing 0.05% Tween20, they were incubated with an anti-Xpress mAb followed by anti-mouse IgG antibody conjugated to HRP specific for naive IgG (Novagen). For Myc-tagged protein blotting, after being blocked, the membrane was incubated with anti-Myc antibody conjugated to HRP (MBL). Each antibody was diluted in Can Get Signal for immunoblotting (TOYOBO). Protein bands were visualized using the Pierce ECL plus Western Blotting Substrate (Thermo), and detected with a LAS4000 instrument (GE HealthCare).

### Statistic analysis

The statistical significance of differences between two groups was determined by the Mann-Whitney U test. A value of p<0.05 was considered to be statistically significant. The numbers of samples are referred to as “n” in each graph.

## Results

### Aid alters the subcellular localization of Tet1 from the nucleus to the cytoplasm

**Figure 1:**
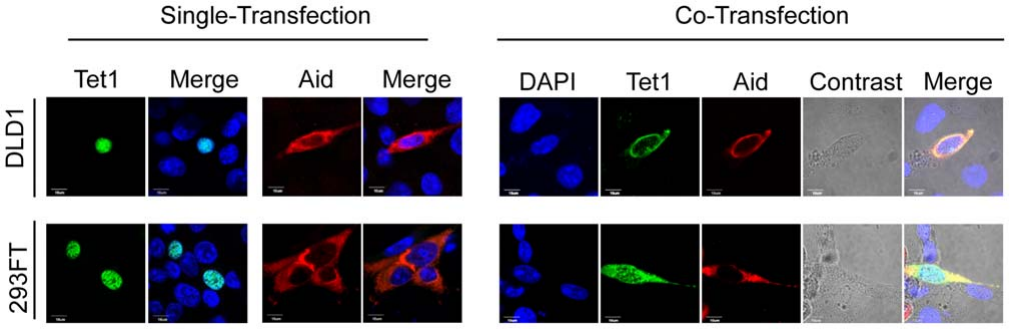
Overexpressed Aid alters the subcellular localization of Tet1. Images of cells transiently expressing N-terminally Xpress-tagged Tet1 or C-terminally Myc-tagged Aid. Tet1 was predominantly localized in the nucleus, whereas Aid was mainly localized in the cytoplasm 48 h after transfection in both DLD-1 and HEK293FT cells. When cells were co-transfected with a plasmid expressing Aid, the Tet1 subcellular localization was altered to the cytoplasm, where Aid was mainly localized. The scale bar is 10 µm.

A previous study showed that Tet family proteins generate 5hmC, whereas Aid facilitates the conversion of 5hmC into cytosine [9]. In this study, we investigated the effect of simultaneous expression of Aid and Tet on their subcellular localization. We transfected C-terminally Myc-tagged Aid or N-terminally Xpress-tagged Tet1 into HEK293FT or DLD-1 cells, and examined the subcellular localization of ectopically expressed proteins. Aid was observed mainly in the cytoplasm, whereas Tet1 was predominantly localized in the nucleus when the single proteins were overexpressed. When cells were co-transfected with expression plasmids for Aid and Tet1, the Tet1 localization was altered from the nucleus to the cytoplasm in the co-transfected cells, whereas Aid remained in the cytoplasm (Fig. 1).

**Figure 2:**
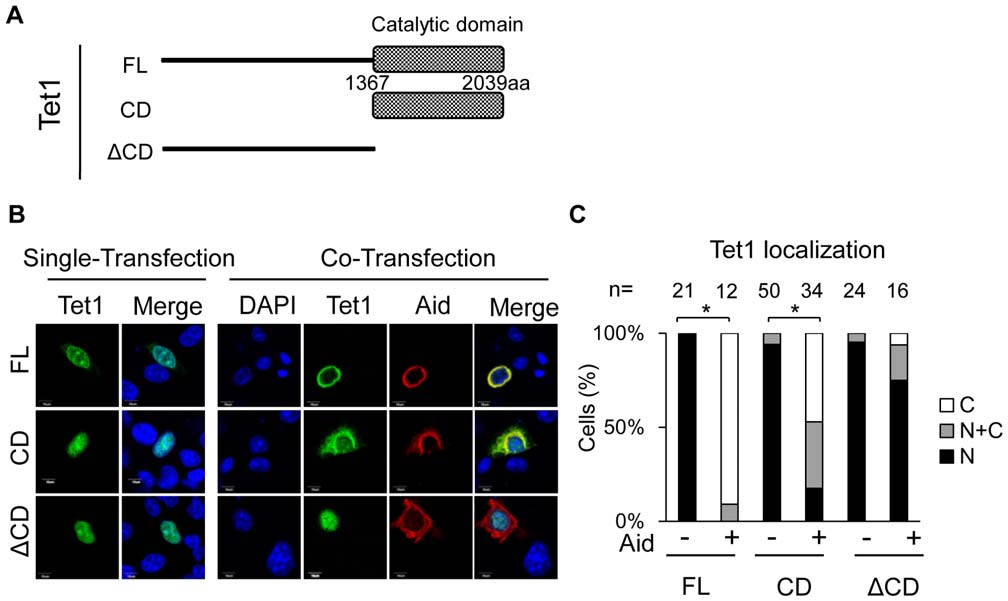
Tet1 translocation requires its catalytic domain. (A) A schematic representation of the Tet1 structure and its mutants used in this study. (aa = amino acid). (B) Confocal images of DLD-1 cells transiently expressing N-terminally Xpress-tagged Tet1 mutants with or without C-terminally Myc-tagged Aid. All Tet1 constructs (FL, CD and ΔCD) were localized in the nucleus when solely expressed in DLD-1 cells. When co-expressed with Aid, Tet1FL and Tet1CD were translocated to the cytoplasm, whereas Tet1ΔCD remained in the nucleus. (C) Each bar represents the proportion of cells with the different localizations of Tet1. The number (n) of cells indicated above each bar was scored according to their subcellular localization. N (black); nuclear localization, N+C (gray); both nuclear and cytoplasmic localization, C (white); cytoplasmic localization in multiple microscope fields. The scale bars in images are 10 µm. *p<0.01.

To determine the domain responsible for the altered localization of Tet1, we performed a subcellular localization analysis using a series of deletion constructs for Tet1, as previously reported [17]; full length (FL) (1–2039 amino acids: aa) which was used in the experiment shown in Fig. 1, the catalytic domain (CD) (1367–2039aa), and the N-terminal domain (ΔCD) (1–1366aa), which lacks CD (Fig. 2A). The Tet1FL plasmid and these mutants were transfected individually with or without the plasmid for Aid. At 48 hrs after transfection, the subcellular localization of Tet1 and Aid was examined by confocal microscopy (Figs. 2B and S2). In the case of single transfection, all Tet1 mutants were predominantly localized in the nucleus, and Aid was mainly localized in the cytoplasm. However, when Tet1FL or Tet1CD was co-expressed with Aid, Tet1 was translocated to the cytoplasm (N: 0%, N+C: 9%, C: 91% for FL, and N: 18%, N+C: 35%, C: 47% for CD, respectively). In contrast, Tet1ΔCD remained in the nucleus even when co-expressed with Aid (N: 75%, N+C: 19%, C: 6%), suggesting that the catalytic domain of Tet1 plays a role in the altered localization of the protein (Figs. 2B and C). Since a previous study indicated that the subcellular localization of Aid is affected by the position of the tag [18], we also carried out co-transfection experiments using untagged Aid protein. It was confirmed that untagged Aid, as well as C-terminal-tagged Aid, also affected the localization of Tet1CD (Fig. S3), supporting the notion that Aid expression alters the subcellular localization of Tet1.

Next, to examine whether this effect is specific to Aid, we carried out the same experiments by using Apobec1 and Apobec2, instead of Aid, both of which show the similar enzymatic activity to Aid [19], [20]. In particular, Apobec1 has been shown to shuttle between the nucleus and the cytoplasm [21]. We observed that overexpressed Apobec1 and Apobec2 were localized at both the nucleus and the cytoplasm in DLD-1 cells regardless of the presence or absence of Tet1CD (Figs. S4). In contrast to the translocation of Tet1CD in the presence of Aid, Tet1CD always remained in the nucleus even when co-expressed with Apobec1 or Apobec2 (Figs. S4B and D, p = 0.11, with vs without Apobec1, p = 0.38, with vs without Apobec2). These results suggest that the altered subcellular localization of Tet1CD is not attributable to the artificial effects due to Aid overexpression.

### Tet1 translocation is independent of its enzymatic activity

**Figure 3:**
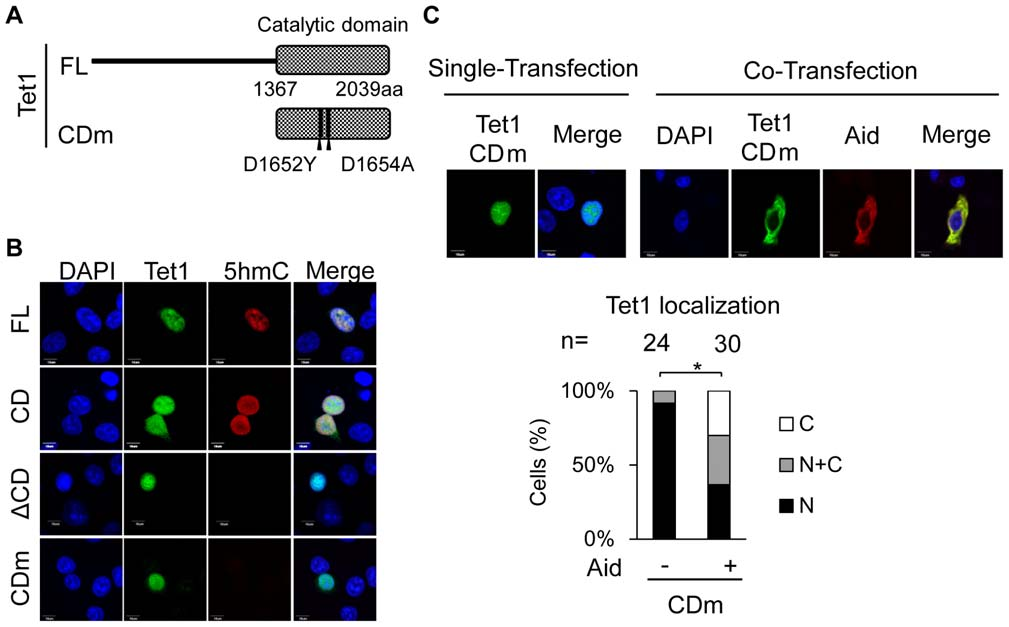
Tet1 translocation in the presence of Aid is independent of the Tet1 enzymatic activity. (A) A schematic representation of the Tet1CD mutant (CDm) used in this study. Tet1CDm were tagged with N-terminal Xpress. (B) Tet1FL and CD had enzyme activity and produced 5hmC, but Tet1ΔCD and CDm did not. (C) C-terminally Myc-tagged Aid expression altered the subcellular localization of Tet1CDm, which lacks the enzymatic activity. The upper panels are representative images of DLD-1 cells transiently expressing Tet1CDm with or without simultaneous expression of Aid. The lower graph shows the percentage score of the examined transfected cells (indicated as a number). The scale bars are 10 µm. *p<0.01. N (black); nuclear localization, N+C (gray); both nuclear and cytoplasmic localization, C (white); cytoplasmic localization in multiple microscope fields.

We observed that the subcellular localization of Tet1 was altered in the presence of Aid, but that Tet1ΔCD remained in the nucleus, implying that Tet1 enzymatic activity for the conversion of 5mC to 5hmC is associated with the translocation of Tet1. To test this, a Tet1CD mutant construct (CDm), which has mutations in the catalytic domain (D1652Y and D1654A) and lacks enzymatic activity, was generated (Fig. 3A) [17]. When Tet1CDm was solely transfected into DLD-1 cells, it was localized in the nucleus. However, the enzyme activity, which was detected by immunostaining for 5hmC, was not observed at all in Tet1CDm-expressing cells while it was evident in Tet1CD- and Tet1 FL-expressing cells (Fig. 3B). We also confirmed that Tet1ΔCD and Aid had no ability to produce 5hmC (Figs. 3B and S5A).

We next examined the subcellular localization of Tet1CDm when it was co-expressed with Aid in DLD-1 cells. Despite the lack of enzymatic activity in Tet1CDm, simultaneous expression of Aid and Tet1CDm caused the altered localization of Tet1CDm, and no significant difference in the localization of Tet1CD and Tet1CDm was observed when they were co-expressed with Aid (p = 0.144, CD vs CDm) (Figs. 2C and 3C). We obtained the similar observation using HEK293FT cells (Fig. S2). These results indicate that, although the catalytic domain of Tet1 is important for the Aid-mediated translocation of Tet1, the translocation occurs independently of its enzymatic activity.

### Co-expression of Aid has similar effects on the subcellular localization of other Tet family proteins

**Figure 4:**
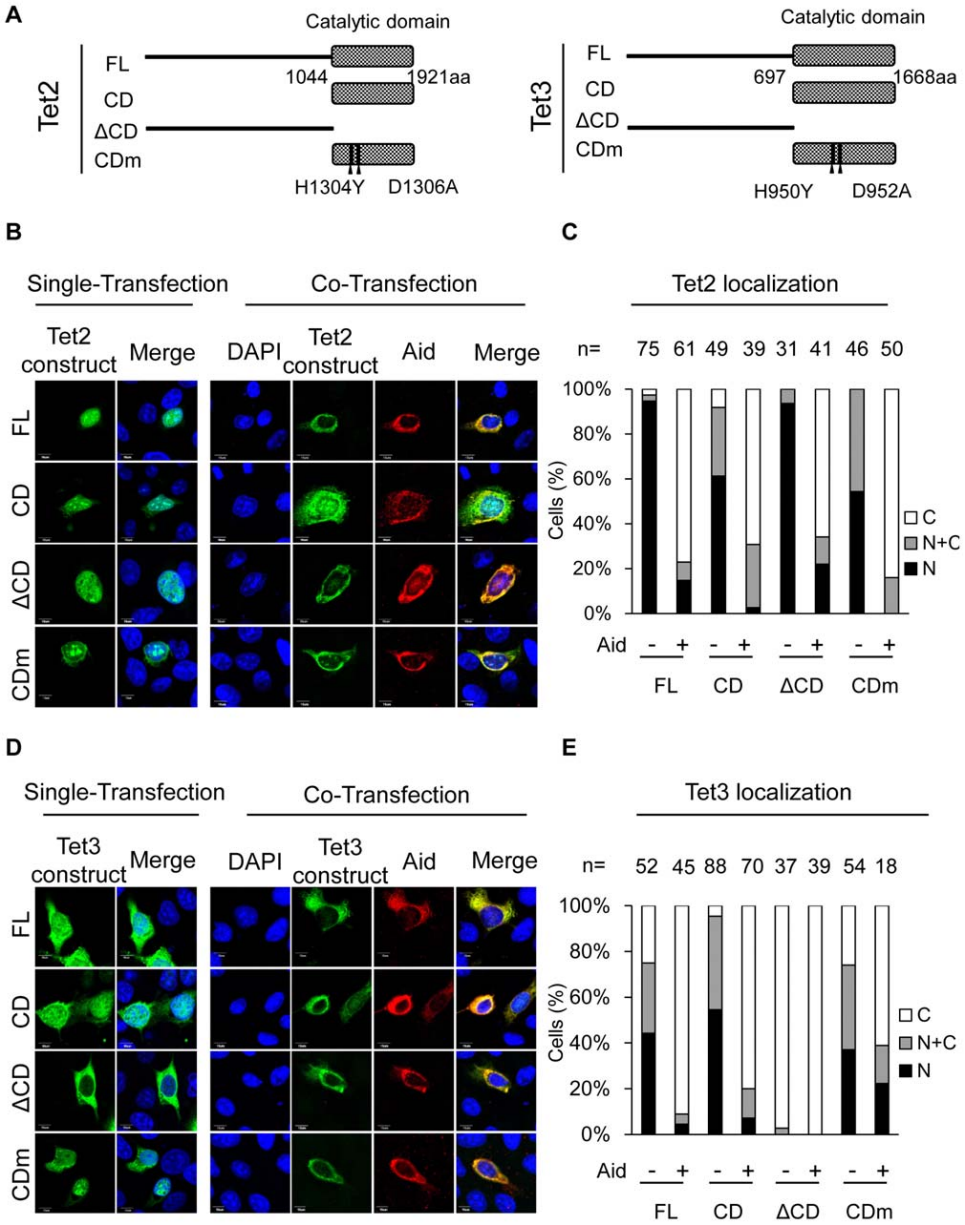
The subcellular localization of Tet2 and Tet3 is altered by Aid expression. (A) A schematic representation of the Tet2 and Tet3 structures and their mutants used in this study. (B) N-terminally Xpress-tagged Tet2 or its mutants with or without Aid tagged with C-terminal Myc were imaged by confocal microscopy in transiently transfected DLD-1 cells. (C) The number (n) of cells indicated above each bar was scored according to Tet2 subcellular localization. All Tet2 mutants were translocated to the cytoplasm in the presence of Aid (p<0.01, vs in the absence of Aid). (D, E) Simultaneous expression of N-terminally Xpress-tagged Tet3 and Aid-Myc. Tet3FL, CD and CDm were translocated to the cytoplasm when co-expressed with Aid (p<0.01, single-expression vs co-expression). Tet3ΔCD was localized in the cytoplasm regardless of the Aid expression. The scale bars are 10 µm. N (black); nuclear localization, N+C (gray); both nuclear and cytoplasmic localization, C (white); cytoplasmic localization in multiple microscope fields.

The Tet family proteins include Tet1, Tet2 and Tet3. We therefore examined whether the effects of Aid co-expression were also observed for other Tet family members. To perform these studies, FL, CD, ΔCD and CDm constructs for both Tet2 and Tet3 were generated (Fig. 4A) based on a previous report [17], and their subcellular localization in the presence or absence of Aid was examined. In both Tet2 and Tet3, the FL and CD proteins exhibited enzymatic activity, whereas the ΔCD and CDm mutants did not (Fig. S5B). Single expression of Tet2FL or its mutants led to the localization of the proteins primarily in the nucleus (Figs. 4B and C). However, in solely Tet3-expressing cells, Tet3ΔCD showed cytoplasmic localization, even in the single transfectants, although Tet3FL, Tet3CD and Tet3CDm were localized in both the nucleus and the cytoplasm (Figs. 4D and E), indicating that the catalytic domain of Tet3 is responsible for the nuclear localization of Tet3. Simultaneous expression of Aid and either of Tet FL, CD or CDm resulted in the altered subcellular localization of both Tet2 and Tet3 into the cytoplasm (Figs. 4B–E). These findings suggest that Aid alters the subcellular localization of all three Tet family proteins, and that this occurs independently of enzyme activity to produce 5hmC.

### Translocation of Tet1 by Aid is associated with Aid shuttling

**Figure 5:**
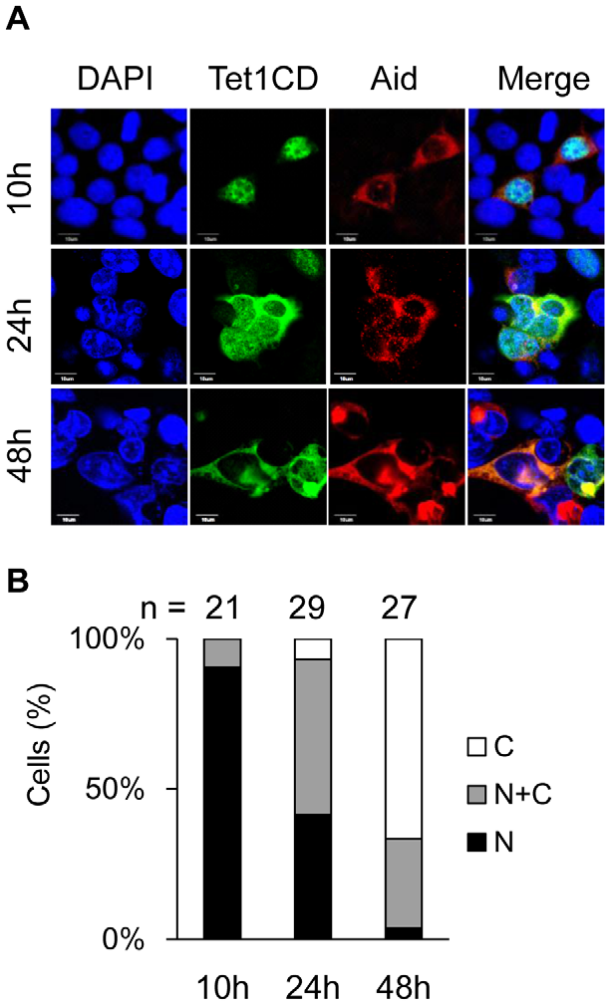
Nuclear Tet1 is gradually translocated into the cytoplasm by the simultaneous expression of Aid. (A) Confocal images of HEK293FT cells transiently co-expressing Tet1CD tagged with N-terminal Xpress and Aid tagged with C-terminal Myc at different time points (10 h, 24 h and 48 h) after co-transfection. The scale bars in images are 10 µm. (B) The number (n) of cells indicated above each bar was scored according to the Tet1CD subcellular localization. The number of cells with cytoplasmic Tet1CD gradually increased after co-transfection. N (black); nuclear localization, N+C (gray); both nuclear and cytoplasmic localization, C (white); cytoplasmic localization in multiple microscope fields.

We next addressed how nuclear Tet1 is translocated into the cytoplasm by Aid. To understand the mechanism, we first examined the localization of both Tet1CD and Aid at different time points (10 h, 24 h, 48 h) after simultaneous transfection into HEK293FT cells. At 10 h after transfection, the subcellular localization of Tet1CD was mainly in the nucleus (N: 90%, N+C: 10%, C: 0%) while Aid was primarily expressed in the cytoplasm, showing the same localization pattern in the single transfected cells. At 24 h after transfection, the proportion of cells with cytoplasmic Tet1CD increased (N: 41%, N+C: 52%, C: 7%), and at 48 h after transfection, most of the Tet1CD were co-localized with Aid in the cytoplasm (N: 3%, N+C: 30%, C: 67%) (Figs. 5A and B). In contrast, Aid could be detected in the cytoplasm throughout this experiment.

**Figure 6:**
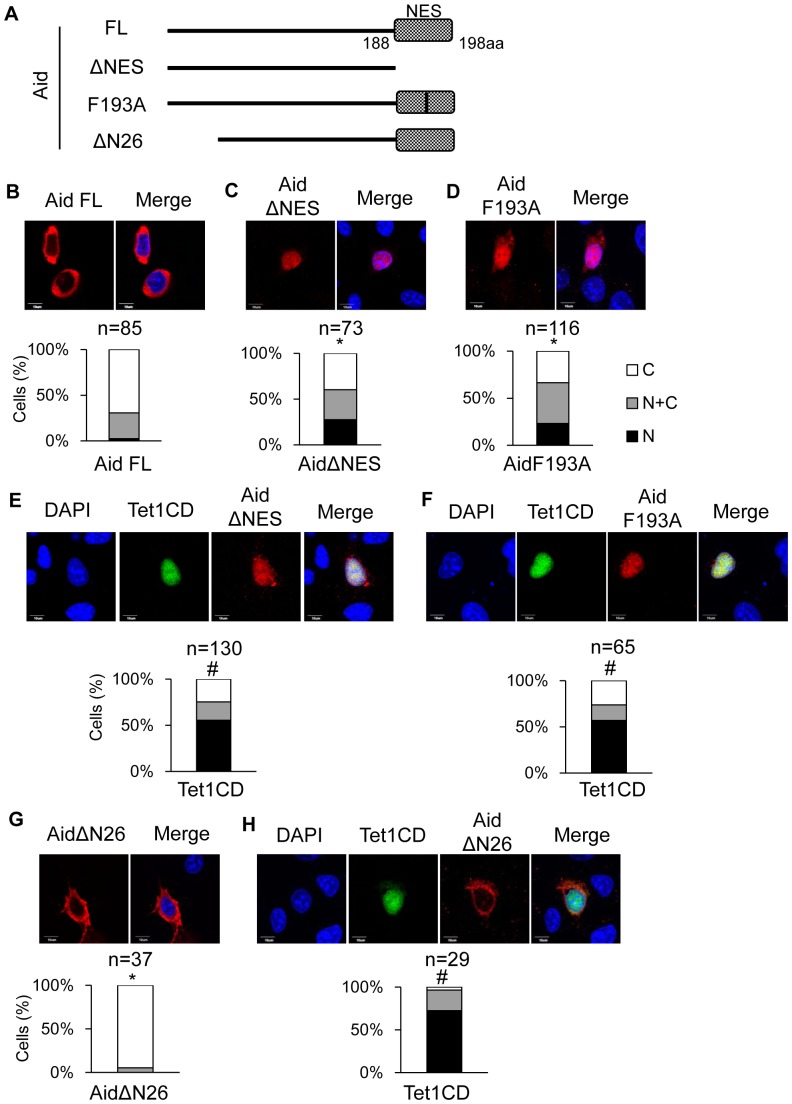
Aid shuttling is associated with Aid-mediating transcloation of Tet1. (A) A schematic representation of the Aid structure and its mutants used in this study. All Aid constructs were tagged with C-terminal Myc. (B–D) The upper figures are representative confocal images of DLD-1 cells transiently expressing only Aid FL (B), ΔNES (C) or F193A (D). The lower figure represents the proportion of cells with different subcellular localization of Aid. Aid mutants defect in NES showed the increased nuclear localization. *p<0.05 vs Aid FL. (E, F) The upper figures are representative confocal images of DLD-1 cells transiently co-expressing AidΔNES and Tet1CD (E), or AidF193A and Tet1CD (F). Tet1CD were tagged with N-terminal Xpress. The lower figure shows the proportion of cells with different localizations of Tet1 (E; AidΔNES and Tet1CD, F; AidF193A and Tet1CD). Aid mutants, which exhibit impaired shuttling between the nucleus and the cytoplasm, failed to alter the subcellular localization of Tet1. #p<0.05 vs with Aid FL. (G) The upper figures are representative confocal images of DLD-1 cells transiently expressing only AidΔN26. The lower figure represents the proportion of cells with different subcellular localization of AidΔN26. *p<0.05 vs Aid FL. (H) The upper figures are representative confocal images of DLD-1 cells transiently co-expressing AidΔN26 and Tet1CD. The lower figure shows the proportion of cells with different localizations of Tet1. #p<0.05 vs with AidFL. The scale bars are 10 µm. N (black); nuclear localization, N+C (gray); both nuclear and cytoplasmic localization, C (white); cytoplasmic localization in multiple microscope fields.

It is worth noting that Aid is a shuttling protein that is translocating between the nucleus and the cytoplasm [12], [13]. Since a gradual increase in the number of cells with cytoplasmic Tet1CD was observed, we evaluated whether Tet1 translocation is associated with Aid shuttling. We performed immunofluorescence experiment by using full length Aid (Aid FL) and its mutants which are impaired in nuclear-cytoplasmic shuttling; Aid lacking NES (AidΔNES_1-187aa), Aid having a single point mutation in the NES (Aid F193A) [22] [23] or Aid lacking the N terminus of Aid, which loses the important sequences for nuclear entry (AidΔN26_27-198aa) [12] (Fig. 6A). As expected, AidΔNES transfected cells revealed an increased number of cells with nuclear localization of Aid (ΔNES; N: 27%, N+C: 33%, C: 40%) when compared with Aid FL- transfected cells (N: 3%, N+C: 28%, C: 69%) (Figs. 6B and C). In addition, Aid F193A showed an increased localization of Aid at the nucleus (N: 23%, N+C: 43%, C: 34%) than Aid FL did. (Figs. 6B and D). Next, we co-transfected these Aid mutants with Tet1CD in DLD-1 cells and examined the effect of the expressions of Aid mutants on the Tet1CD subcellular localization (Figs. 6E and F). Co-expression with AidΔNES resulted in a decrease in the number of Tet1CD-translocated cells (N: 55%, N+C: 20%, C: 25%) compared with that induced by with Aid FL (N: 18%, N+C: 35%, C: 47%) (Figs. 2C and 6E). Similarly, when co-expressed with a single point mutant AidF193A, the proportion of Tet1CD-translocated cells were decreased (N: 57%, N+C: 17%, C: 26%) in comparison to those when co-transfected with Aid FL (Figs. 2C and 6F). In addition, we performed the similar experiment using AidΔN26, which has defect in nuclear entry. AidΔN26 was predominantly localized at the cytoplasm in the case of single expression (N: 0%, N+C: 5%, C: 95%) (Fig. 6G). When co-expressed with AidΔN26, Tet1CD remained in the nucleus (N: 72%, N+C: 24%, C: 4%) (Figs. 2C and 6H). Taken together, these findings imply that Aid shuttling, which is mediated by the N-terminus and C-terminus domains of Aid, is associated with the Aid-mediated translocation of Tet1, and suggest that Tet1 translocation is dependent on the subcellular localization of Aid.

### Interaction between Aid and Tet1

**Figure 7:**
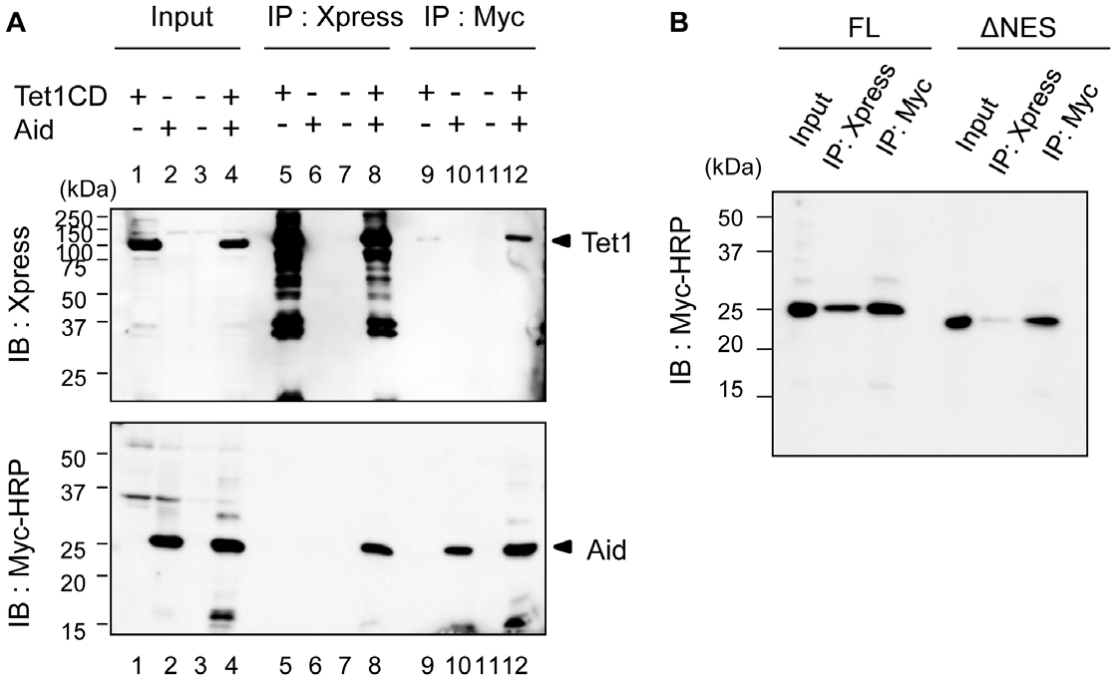
Aid interacts with Tet1CD. (A) Tet1CD was co-immunoprecipitated with Aid FL. Lysates from HEK293FT cells transfected with N-terminally Xpress-tagged Tet1CD, C-terminally Myc-tagged AidFL or both of them were immunoprecipitated (IP) by anti-Xpress mAbs or anti-Myc mAbs. Immunoblotting (IB) was performed by using an anti-Xpress Abs or anti-Myc-HRP antibody. Lane nos. 1, 5 and 9 were single transfections of Tet1CD. Lane nos. 2, 6 and 10 were single-transfections of Aid. Lane nos. 3, 7 and 11 were for mock transfection. Lane nos. 4, 8 and 12 shows the results for the co-transfection of Tet1CD and Aid. (B) The Co-IP experiment was performed by using lysates from HEK293FT cells co-transfected with N-terminally Xpress tagged-Tet1CD and C-terminally Myc-tagged Aid FL, or with N-terminally Xpress-tagged Tet1CD and C-terminally Myc-tagged AidΔNES. Despite the similar localization of AidΔNES and Tet1CD in the nucleus, AidΔNES revealed a decreased association with Tet1CD compared to Aid FL.

In this study, we found that nuclear Tet1 is translocated to the cytoplasm by Aid, and that the translocated Tet1 is co-localized with Aid. We next examined whether Aid interacts with Tet1 during this Aid-mediated translocation of Tet1. We carried out co-immunoprecipitation (co-IP) – immunoblotting (IB) using HEK293FT cells transfected with either or both the Xpress-Tet1CD and Aid FL-Myc vectors. Empty vectors were used as a negative control. Aid FL-Myc was co-precipitated with an anti-Xpress mAbs for Xpress-Tet1CD, and this association was confirmed by reciprocal IP with anti-Myc mAbs (Fig. 7A). Moreover, we observed a decreased interaction of AidΔNES with Tet1CD, although both AidΔNES and Tet1CD were localized in the nucleus (Fig. 7B). This result indicates that the NES domain of Aid is associated with the interaction between Aid and Tet1.

## Discussion

Aid shuttles between the nucleus and the cytoplasm, interacting with several molecules, such as RNA polymerase II [24], CTNNBL-1 [25] and GANP [26], in order to target the IgV region and/or the S region DNA. Previous studies proposed that the shuttling of Aid plays a role in preventing excessive DNA mutation in the nucleus [18], [27]. In the present study, we showed that the simultaneous expression of Aid and Tet family enzymes causes the altered subcellular localization of the Tet family proteins. Furthermore, the translocation of Tet was affected by Aid shuttling between the nucleus and the cytoplasm. These results suggest that Aid shuttling might have another function; altering the subcellular localization of Tet family members. However, it should be also noted that the level of Aid induced in this experiments seems to be substantially higher than that of physiological condition. Considering such artificial experimental system, further analyses for endogenous proteins are required to conclude the physiological function of Aid in the translocation of Tet family enzymes.

Although the physiological relevance of our findings remains to be established, it is important to note that the expression of both Tet family proteins and Aid is restricted to specific cell types. It was reported that Aid is highly expressed in oocytes [28], while Tet3 is expressed at high levels in oocytes and zygotes [6], thus indicating that both Tet3 and Aid are abundantly expressed in oocytes. Of note, Tet3 is localized in the cytoplasm in oocytes, but it translocates into the male pronucleus of zygotes shortly after fertilization [16]. Therefore, it seems that there is dynamic regulation of the subcellular localization of Tet family members during the early stage of development. Considering that simultaneous expression of Aid and Tet family members caused the translocalization of Tet proteins into the cytoplasm in this study, it is possible that endogenously expressed Aid contributes to the cytoplasmic localization of Tet3 in oocytes.

DNA methylation is critical for mammalian development and cellular differentiation [29]. In mammals, active genomic demethylation contributes to the genome-wide erasure of the DNA methylation observed in preimplantation embryos and primordial germ cells (PGCs) [30], [31]. However, the mechanisms underlying active DNA demethylation in mammals have been highly controversial, although multiple mechanisms have been proposed [32]–[34]. Recently, an additional model was reported, wherein Aid facilitates the conversion of 5hmC into cytosine [9], [35], and forms several complexes with thymine DNA glycosylase and GADD45a, which are involved in active DNA demethylation [36]. Our findings may also support the notion that Aid plays a role in DNA demethylation while interacting with several related factors.

To determine whether the altered subcellular localization of Tet contributes to the altered production of 5hmC, we performed immunodetection for 5hmC in dually-transfected cells (expressing both Tet1 and Aid), where Tet1 was translocated into the cytoplasm. Although Tet1 CD had already been translocated into the cytoplasm, 5hmC was still detectable in the nucleus (Fig. S6). Therefore, we could not conclude whether the translocation of Tet can affect the production of 5hmC in cells expressing both proteins. One of the possible explanations for our observation is that 5hmC is, to some extent, stable after its production, which made it difficult to detect an alteration in the 5hmC levels under our experimental conditions.

In summary, our present findings indicate that Aid regulates the subcellular localization of Tet family proteins, and that this is associated with Aid shuttling. The subcellular localization of proteins is crucial for their functional activity and is associated with their functional diversity [37], [38]. Since both Aid and the Tet family proteins are involved in the modification of 5hmC, the coordinated action of these proteins might control epigenetic modifications by affecting the subcellular localization of Tet family proteins as we described in this study. Further studies are warranted to uncover the functional and physiological significance of the Aid-mediated translocation of Tet, which may eventually extend our understanding of the regulation of 5hmC production and active DNA demethylation.

## Supporting Information

Figure S1 **Representative images for each subcellular localization.** Representative images of each subcellular localization are shown. Dominant immunofluorescent signals in the nucleus and cytoplasm were regarded as nuclear localization (N) and cytoplasmic localization (C), respectively. Similar signal intensity in both the nucleus and the cytoplasm was regarded as C+N. Scale bars are 10 µm.
(TIF)

Figure S2 **Aid alters the subcellular localization of Tet1 in HEK293FT cells.** HEK293FT cells expressing Aid also revealed translocation of Tet1CD and CDm. Consistent with the results in DLD-1 cells, Tet1ΔCD was retained in the nucleus even in the presence of Aid. The scale bars are 10 µm.
(TIF)

Figure S3 **Untagged Aid expression results in the subcellular translocation of Tet1.** Untagged Aid was detected by an anti-Aid polyclonal antibody. Untagged Aid was mainly localized in the cytoplasm, which was the same as Myc-tagged Aid. Simultaneous expression of untagged Aid and Tet1CD caused the altered localization of Tet1CD in the cytoplasm. The scale bars are 10 µm.
(TIF)

Figure S4 **Apobec family has not an effect on the subcellular localization of Tet1.** (A) The upper figures were confocal images of DLD-1 cells transiently expressing C-terminally Myc-tagged Apobec1. The lower graph represents the proportion of cells with different subcellular localization of Apobec1. (B) The upper figures were images of DLD-1 cells transiently co-expressing C-terminally Myc-tagged Apobec1 and N-terminally Xpress-tagged Tet1CD. The lower graph represents the percentage of the different subcellular localization of Tet1CD on the co-expressing cells. (C) The upper was images of DLD-1 cells transiently expressing C-terminally Myc-tagged Apobec2. The lower represents the proportion of cells with different subcellular localization of Apobec2. (D) The upper were images of DLD-1 cells transiently co-expressing C-terminally Myc-tagged Apobec2 and N-terminally Xpress-tagged Tet1CD. The lower showed the proportion of cells with different subcellular localization of Tet1CD on the co-expressing cells. The scale bars are 10 µm. N (black); nuclear localization, N+C (gray); both nuclear and cytoplasmic localization, C (white); cytoplasmic localization in multiple microscope fields.
(TIF)

Figure S5 **Detection of 5hmC by immunostaining using in DLD-1 cells.** (A) Aid alone could not produce 5hmC. (B) The FL and CD had enzymatic activity, whereas the ΔCD and CDm proteins did not in both Tet2 and Tet3. Aid was tagged with C-terminal Myc and Tets were with N-terminal Xpress. The scale bars are 10 µm.
(TIF)

Figure S6 **5hmC remains in the nucleus even after Tet1CD transfer to the cytoplasm in HEK293FT cells.** The 5hmC could be still detected in the nucleus, even though Tet1CD was translocated from the nucleus to the cytoplasm in the presence of Aid. Aid was tagged with C-terminal Myc and Tets were with N-terminal Xpress. The scale bars are 20 µm.
(TIF)

Table S1 **Primer sets for cloning Tet family and Aid used in this study.** F: forward primer, R: reverse primer
(DOC)

## References

[1] Bird A (2002) DNA methylation patterns and epigenetic memory. Genes Dev 16: 6–21. 11782440 10.1101/gad.947102

[2] Goll MG, Bestor TH (2005) Eukaryotic cytosine methyltransferases. Annu Rev Biochem 74: 481–514. 15952895 10.1146/annurev.biochem.74.010904.153721

[3] Chen T, Li E (2004) Structure and function of eukaryotic DNA methyltransferases. Curr Top Dev Biol 60: 55–89. 15094296 10.1016/S0070-2153(04)60003-2

[4] Reik W, Dean W, Walter J (2001) Epigenetic reprogramming in mammalian development. Science 293: 1089–1093. 11498579 10.1126/science.1063443

[5] Inoue A, Zhang Y (2011) Replication-dependent loss of 5-hydroxymethylcytosine in mouse preimplantation embryos. Science 334: 194. 21940858 10.1126/science.1212483 PMC3799877

[6] Iqbal K, Jin SG, Pfeifer GP, Szabó PE (2011) Reprogramming of the paternal genome upon fertilization involves genome-wide oxidation of 5-methylcytosine. Proc Natl Acad Sci U S A 108: 3642–3647. 21321204 10.1073/pnas.1014033108 PMC3048122

[7] Wossidlo M, Nakamura T, Lepikhov K, Marques CJ, Zakhartchenko V, et al (2011) 5-Hydroxymethylcytosine in the mammalian zygote is linked with epigenetic reprogramming. Nat Commun 2: 241. 21407207 10.1038/ncomms1240

[8] Tahiliani M, Koh KP, Shen Y, Pastor WA, Bandukwala H, et al (2009) Conversion of 5-methylcytosine to 5-hydroxymethylcytosine in mammalian DNA by MLL partner TET1. Science 324: 930–935. 19372391 10.1126/science.1170116 PMC2715015

[9] Guo JU, Su Y, Zhong C, Ming GL, Song H (2011) Hydroxylation of 5-methylcytosine by TET1 promotes active DNA demethylation in the adult brain. Cell 145: 423–434. 21496894 10.1016/j.cell.2011.03.022 PMC3088758

[10] Di Noia JM, Neuberger MS (2007) Molecular mechanisms of antibody somatic hypermutation. Annu Rev Biochem 76: 1–22. 17328676 10.1146/annurev.biochem.76.061705.090740

[11] Muramatsu M, Kinoshita K, Fagarasan S, Yamada S, Shinkai Y, et al (2000) Class switch recombination and hypermutation require activation-induced cytidine deaminase (AID), a potential RNA editing enzyme. Cell 102: 553–563. 11007474 10.1016/s0092-8674(00)00078-7

[12] Ito S, Nagaoka H, Shinkura R, Begum N, Muramatsu M, et al (2004) Activation-induced cytidine deaminase shuttles between nucleus and cytoplasm like apolipoprotein B mRNA editing catalytic polypeptide 1. Proc Natl Acad Sci U S A 101: 1975–1980. 14769937 10.1073/pnas.0307335101 PMC357037

[13] Patenaude AM, Di Noia JM (2010) The mechanisms regulating the subcellular localization of AID. Nucleus 1: 325–331. 21327080 10.4161/nucl.1.4.12107 PMC3027040

[14] Rada C, Jarvis JM, Milstein C (2002) AID-GFP chimeric protein increases hypermutation of Ig genes with no evidence of nuclear localization. Proc Natl Acad Sci U S A 99: 7003–7008. 12011459 10.1073/pnas.092160999 PMC124518

[15] Yang G, Obiakor H, Sinha RK, Newman BA, Hood BL, et al (2005) Activation-induced deaminase cloning, localization, and protein extraction from young VH-mutant rabbit appendix. Proc Natl Acad Sci U S A 102: 17083–17088. 16280388 10.1073/pnas.0501338102 PMC1282565

[16] Gu TP, Guo F, Yang H, Wu HP, Xu GF, et al (2011) The role of Tet3 DNA dioxygenase in epigenetic reprogramming by oocytes. Nature 477: 606–610. 21892189 10.1038/nature10443

[17] Ito S, D'Alessio AC, Taranova OV, Hong K, Sowers LC, et al (2010) Role of Tet proteins in 5mC to 5hmC conversion, ES-cell self-renewal and inner cell mass specification. Nature 466: 1129–1133. 20639862 10.1038/nature09303 PMC3491567

[18] Patenaude AM, Orthwein A, Hu Y, Campo VA, Kavli B, et al (2009) Active nuclear import and cytoplasmic retention of activation-induced deaminase. Nat Struct Mol Biol 16: 517–527. 19412186 10.1038/nsmb.1598

[19] Conticello SG (2008) The AID/APOBEC family of nucleic acid mutators. Genome Biol 9: 229. 10.1186/gb-2008-9-6-229 PMC2481415 18598372

[20] Navaratnam N, Sarwar R (2006) An overview of cytidine deaminases. Int J Hematol 83: 195–200. 16720547 10.1532/IJH97.06032

[21] Chester A, Somasekaram A, Tzimina M, Jarmuz A, Gisbourne J, et al (2003) The apolipoprotein B mRNA editing complex performs a multifunctional cycle and suppresses nonsense-mediated decay. EMBO J 22: 3971–3982. 12881431 10.1093/emboj/cdg369 PMC169042

[22] McBride KM, Barreto V, Ramiro AR, Stavropoulos P, Nussenzweig MC (2004) Somatic hypermutation is limited by CRM1-dependent nuclear export of activation-induced deaminase. J Exp Med 199: 1235–1244. 15117971 10.1084/jem.20040373 PMC2211910

[23] Geisberger R, Rada C, Neuberger MS (2009) The stability of AID and its function in class-switching are critically sensitive to the identity of its nuclear-export sequence. Proc Natl Acad Sci U S A 106: 6736–6741. 19351893 10.1073/pnas.0810808106 PMC2672500

[24] Nambu Y, Sugai M, Gonda H, Lee CG, Katakai T, et al (2003) Transcription-coupled events associating with immunoglobulin switch region chromatin. Science 302: 2137–2140. 14684824 10.1126/science.1092481

[25] Conticello SG, Ganesh K, Xue K, Lu M, Rada C, et al (2008) Interaction between antibody-diversification enzyme AID and spliceosome-associated factor CTNNBL1. Mol Cell 31: 474–484. 18722174 10.1016/j.molcel.2008.07.009

[26] Maeda K, Singh SK, Eda K, Kitabatake M, Pham P, et al (2010) GANP-mediated recruitment of activation-induced cytidine deaminase to cell nuclei and to immunoglobulin variable region DNA. J Biol Chem 285: 23945–23953. 20507984 10.1074/jbc.M110.131441 PMC2911284

[27] Stavnezer J (2011) Complex regulation and function of activation-induced cytidine deaminase. Trends Immunol 32: 194–201. 21493144 10.1016/j.it.2011.03.003 PMC3090464

[28] Morgan HD, Dean W, Coker HA, Reik W, Petersen-Mahrt SK (2004) Activation-induced cytidine deaminase deaminates 5-methylcytosine in DNA and is expressed in pluripotent tissues: implications for epigenetic reprogramming. J Biol Chem 279: 52353–52360. 15448152 10.1074/jbc.M407695200

[29] Sasaki H, Matsui Y (2008) Epigenetic events in mammalian germ-cell development: reprogramming and beyond. Nat Rev Genet 9: 129–140. 18197165 10.1038/nrg2295

[30] Guibert S, Forné T, Weber M (2012) Global profiling of DNA methylation erasure in mouse primordial germ cells. Genome Res 10.1101/gr.130997.111 PMC3317146 22357612

[31] Zaitseva I, Zaitsev S, Alenina N, Bader M, Krivokharchenko A (2007) Dynamics of DNA-demethylation in early mouse and rat embryos developed in vivo and in vitro. Mol Reprod Dev 74: 1255–1261. 17290422 10.1002/mrd.20704

[32] Rai K, Huggins IJ, James SR, Karpf AR, Jones DA, et al (2008) DNA demethylation in zebrafish involves the coupling of a deaminase, a glycosylase, and gadd45. Cell 135: 1201–1212. 19109892 10.1016/j.cell.2008.11.042 PMC2629358

[33] Bhutani N, Brady JJ, Damian M, Sacco A, Corbel SY, et al (2010) Reprogramming towards pluripotency requires AID-dependent DNA demethylation. Nature 463: 1042–1047. 20027182 10.1038/nature08752 PMC2906123

[34] Popp C, Dean W, Feng S, Cokus SJ, Andrews S, et al (2010) Genome-wide erasure of DNA methylation in mouse primordial germ cells is affected by AID deficiency. Nature 463: 1101–1105. 20098412 10.1038/nature08829 PMC2965733

[35] Bhutani N, Burns DM, Blau HM (2011) DNA demethylation dynamics. Cell 146: 866–872. 21925312 10.1016/j.cell.2011.08.042 PMC3236603

[36] Cortellino S, Xu J, Sannai M, Moore R, Caretti E, et al (2011) Thymine DNA glycosylase is essential for active DNA demethylation by linked deamination-base excision repair. Cell 146: 67–79. 21722948 10.1016/j.cell.2011.06.020 PMC3230223

[37] Hung MC, Link W (2011) Protein localization in disease and therapy. J Cell Sci 124: 3381–3392. 22010196 10.1242/jcs.089110

[38] Butler GS, Overall CM (2009) Proteomic identification of multitasking proteins in unexpected locations complicates drug targeting. Nat Rev Drug Discov 8: 935–948. 19949400 10.1038/nrd2945

