# Supplementary figures and images for: Activation-Induced Cytidine Deaminase Alters the Subcellular Localization of Tet Family Proteins
Source: PLoS One. 2012 Sep 17;7(9):e45031. doi: 10.1371/journal.pone.0045031 (PMC3444495; doi:10.1371/journal.pone.0045031)

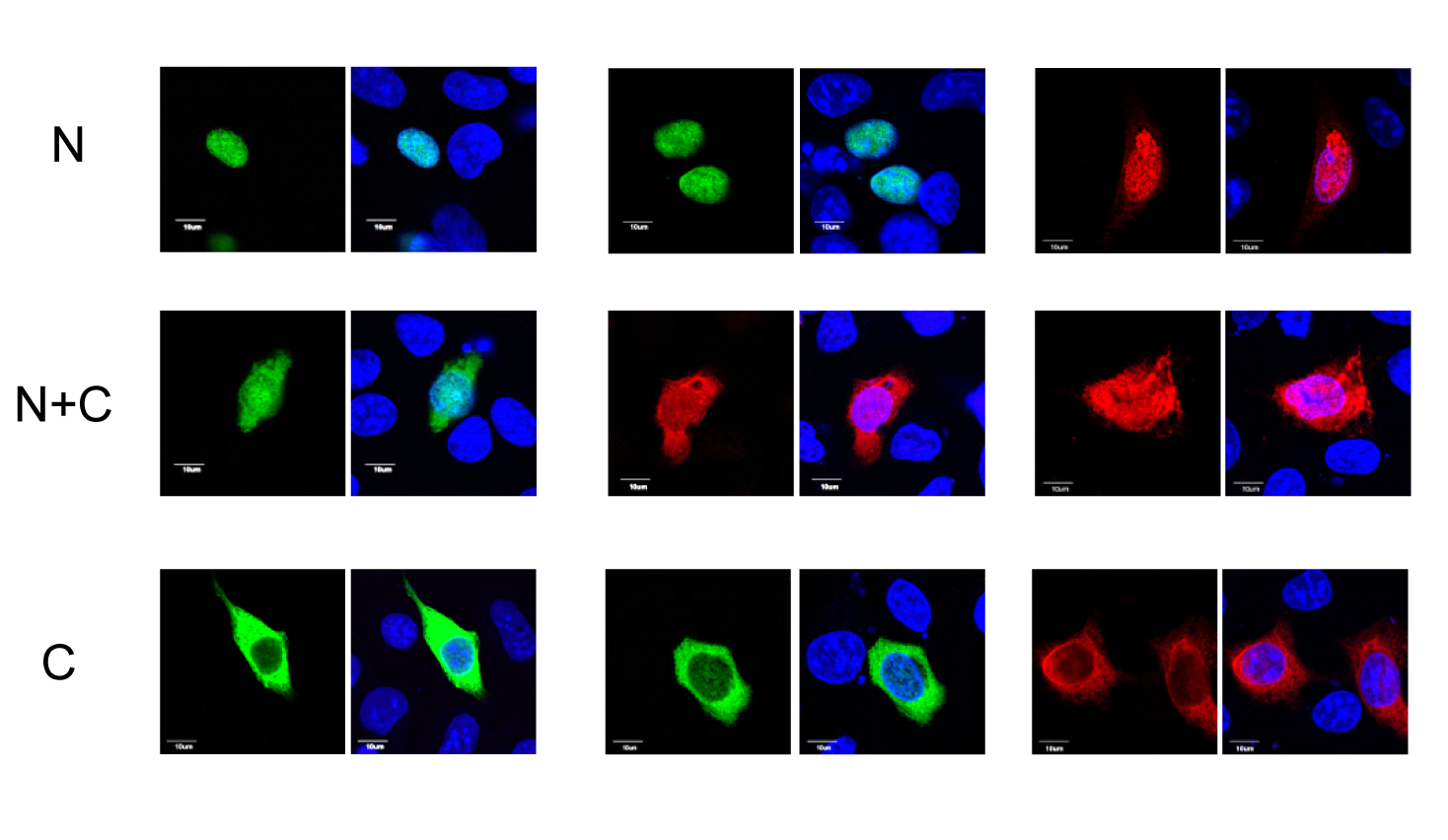

Supplement: Figure S1 — Representative images for each subcellular localization. Representative images of each subcellular localization are shown. Dominant immunofluorescent signals in the nucleus and cytoplasm were regarded as nuclear localization (N) and cytoplasmic localization (C), respectively. Similar signal intensity in both the nucleus and the cytoplasm was regarded as C+N. Scale bars are 10 µm. (TIF) [file pone.0045031.s001.tif]

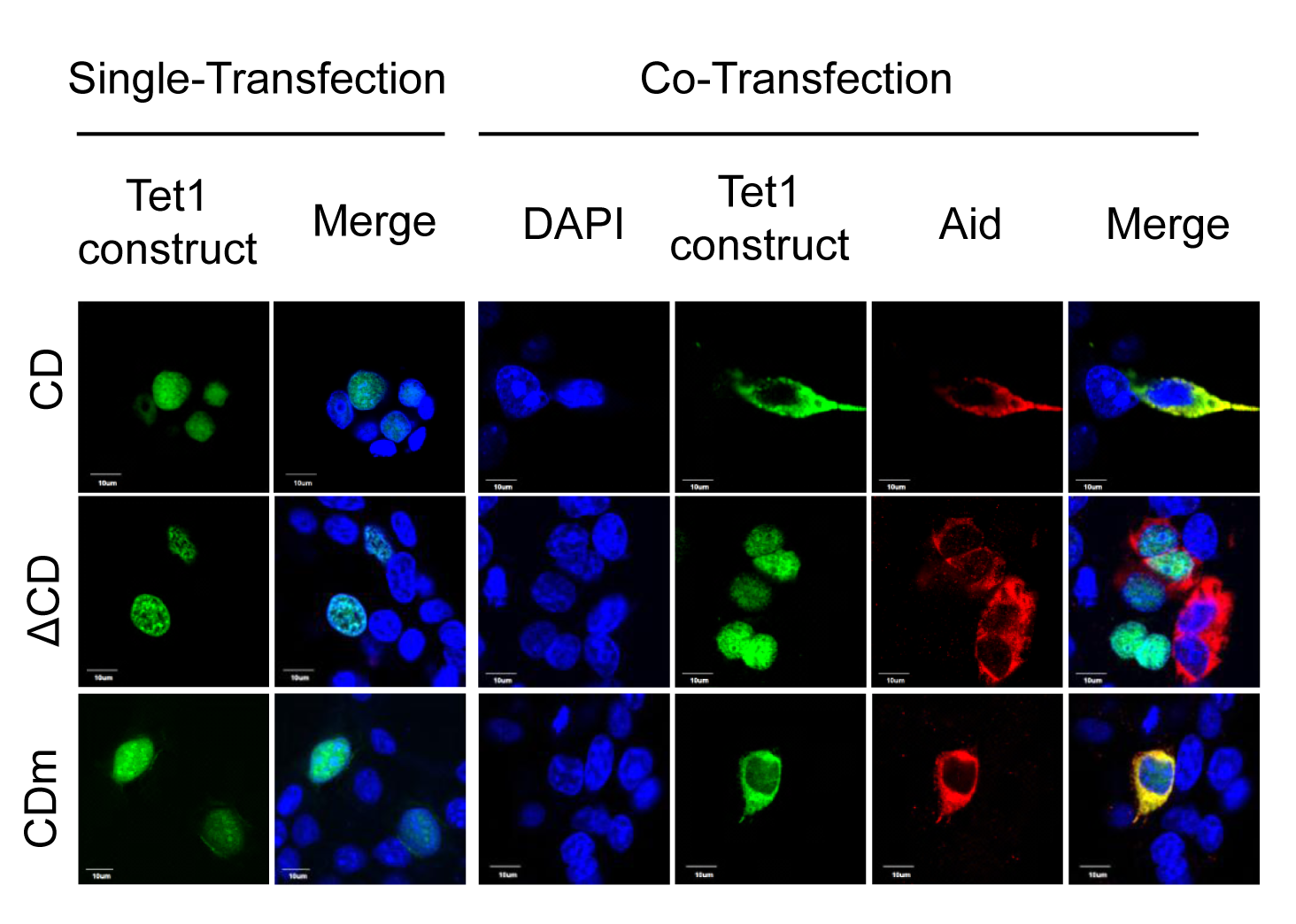

Supplement: Figure S2 — Aid alters the subcellular localization of Tet1 in HEK293FT cells. HEK293FT cells expressing Aid also revealed translocation of Tet1CD and CDm. Consistent with the results in DLD-1 cells, Tet1ΔCD was retained in the nucleus even in the presence of Aid. The scale bars are 10 µm. (TIF) [file pone.0045031.s002.tif]

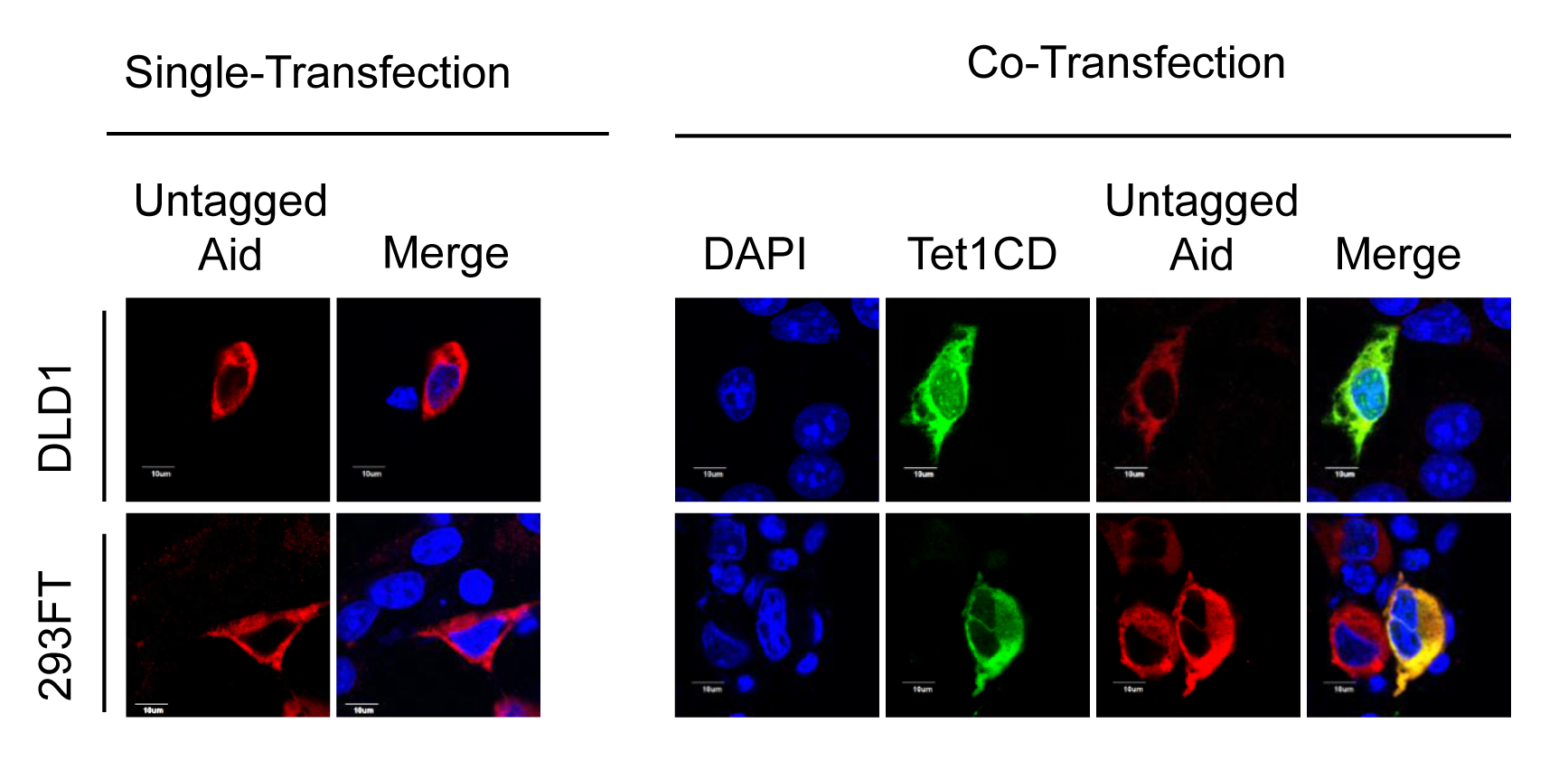

Supplement: Figure S3 — Untagged Aid expression results in the subcellular translocation of Tet1. Untagged Aid was detected by an anti-Aid polyclonal antibody. Untagged Aid was mainly localized in the cytoplasm, which was the same as Myc-tagged Aid. Simultaneous expression of untagged Aid and Tet1CD caused the altered localization of Tet1CD in the cytoplasm. The scale bars are 10 µm. (TIF) [file pone.0045031.s003.tif]

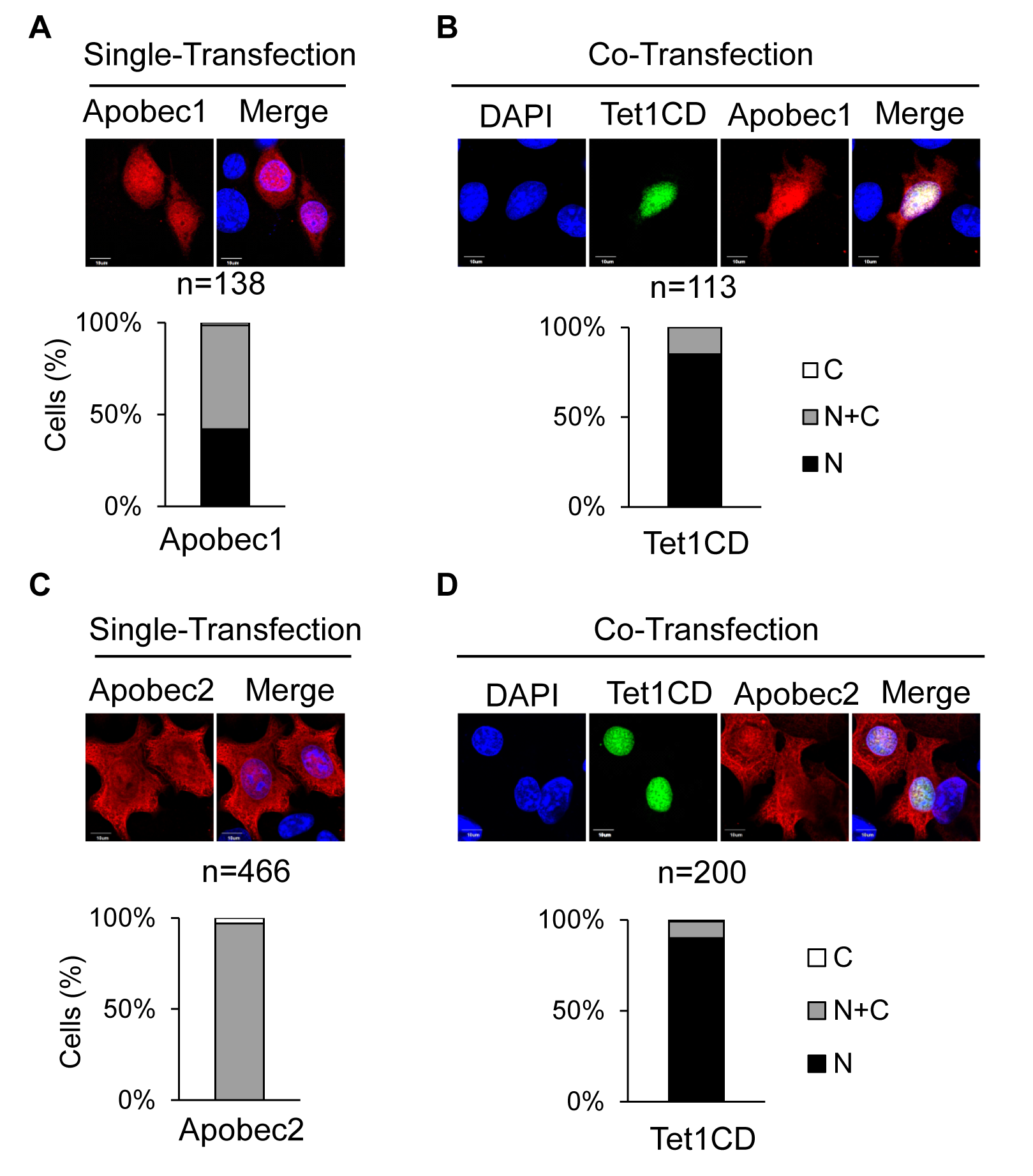

Supplement: Figure S4 — Apobec family has not an effect on the subcellular localization of Tet1. (A) The upper figures were confocal images of DLD-1 cells transiently expressing C-terminally Myc-tagged Apobec1. The lower graph represents the proportion of cells with different subcellular localization of Apobec1. (B) The upper figures were images of DLD-1 cells transiently co-expressing C-terminally Myc-tagged Apobec1 and N-terminally Xpress-tagged Tet1CD. The lower graph represents the percentage of the different subcellular localization of Tet1CD on the co-expressing cells. (C) The upper was images of DLD-1 cells transiently expressing C-terminally Myc-tagged Apobec2. The lower represents the proportion of cells with different subcellular localization of Apobec2. (D) The upper were images of DLD-1 cells transiently co-expressing C-terminally Myc-tagged Apobec2 and N-terminally Xpress-tagged Tet1CD. The lower showed the proportion of cells with different subcellular localization of Tet1CD on the co-expressing cells. The scale bars are 10 µm. N (black); nuclear localization, N+C (gray); both nuclear and cytoplasmic localization, C (white); cytoplasmic localization in multiple microscope fields. (TIF) [file pone.0045031.s004.tif]

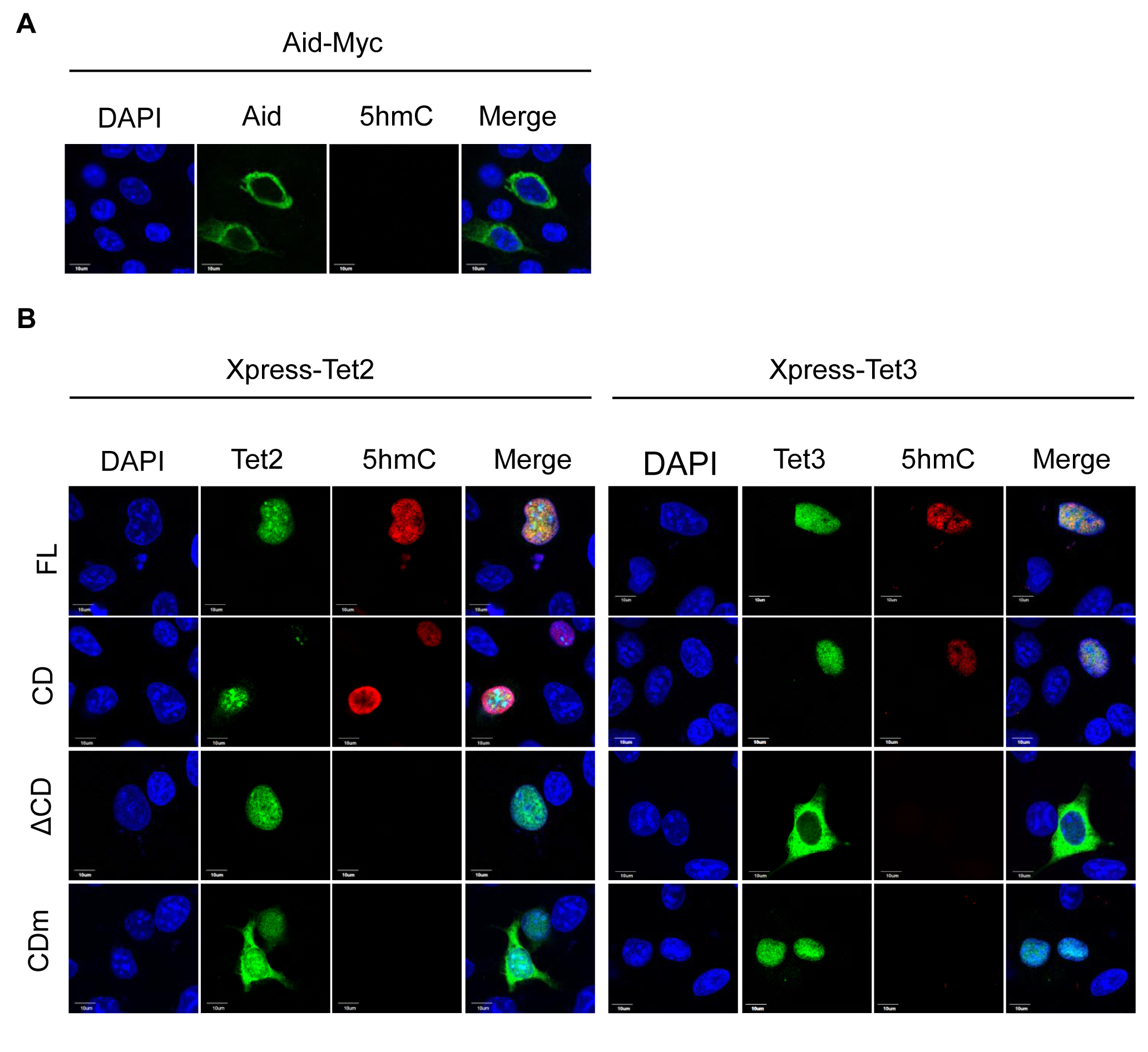

Supplement: Figure S5 — Detection of 5hmC by immunostaining using in DLD-1 cells. (A) Aid alone could not produce 5hmC. (B) The FL and CD had enzymatic activity, whereas the ΔCD and CDm proteins did not in both Tet2 and Tet3. Aid was tagged with C-terminal Myc and Tets were with N-terminal Xpress. The scale bars are 10 µm. (TIF) [file pone.0045031.s005.tif]

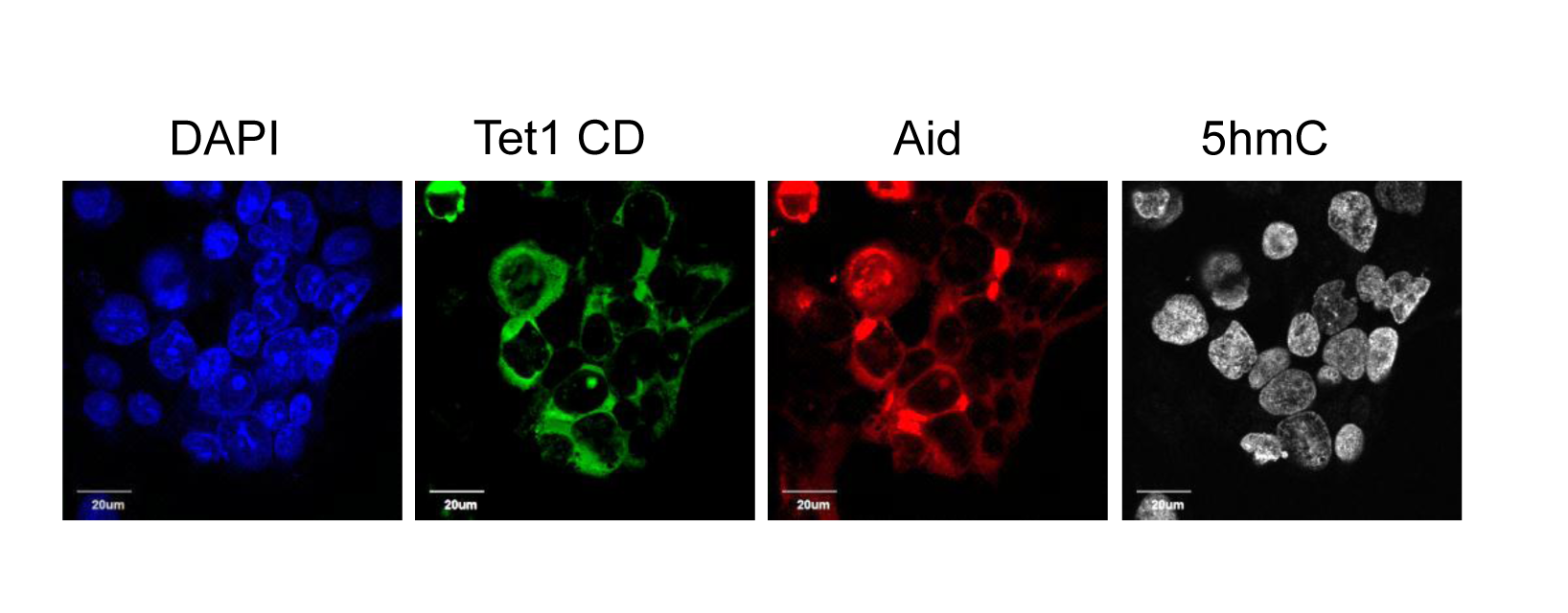

Supplement: Figure S6 — 5hmC remains in the nucleus even after Tet1CD transfer to the cytoplasm in HEK293FT cells. The 5hmC could be still detected in the nucleus, even though Tet1CD was translocated from the nucleus to the cytoplasm in the presence of Aid. Aid was tagged with C-terminal Myc and Tets were with N-terminal Xpress. The scale bars are 20 µm. (TIF) [file pone.0045031.s006.tif]
