# Supplementary material for: Activation-Induced Cytidine Deaminase Alters the Subcellular Localization of Tet Family Proteins
Source: PLoS One. 2012 Sep 17;7(9):e45031. doi: 10.1371/journal.pone.0045031 (PMC3444495; doi:10.1371/journal.pone.0045031)
Supplement: Table S1 — Primer sets for cloning Tet family and Aid used in this study. F: forward primer, R: reverse primer (DOC) [file pone.0045031.s007.doc]

**Table S1. Primer sets for cloning Tet family and Aid used in this study.**

| gene |  | Sequence 5’→3’ | restriction enzyme |
| --- | --- | --- | --- |
| Tet1 | F | GGATCCTCTCGGTCCCGCCCCGCAAAG | Bam |
|  | R | GCGGCCGCTTAGACCCAACGATTGTAGGGTC | Not |
| Tet2 | F | GAATTCGAACAGGACAGAACCACCCATG | EcoRI |
|  | R | CTCGAGTCATACAAATGTGTTGTAAGGCC | XhoI |
| Tet3 | F | GAATTCGACTCAGGGCCAGTGTACCATG | EcoRI |
|  | R | GCGGCCGCCTAGATCCAGCGGCTGTAGG | NotI |
| Aid | F | GGATCCACCATGGACAGCCTTCTGATGAAGC | BamHI |
|  | R | CTCGAGAAATCCCAACATACGAAATGCATC | XhoI |
| Apobec1 | F | GGATCCACCATGAGTTCCGAGACAGGCCC | BamHI |
|  | R | CTCGAGTTTCAACCCTGTAGCCCAAAGG | XhoI |
| Apobec2 | F | GGATCCACCATGGCTCAGAAGGAAGAGGC | BamHI |
|  | R | CTCGAGCTTCAGGATGTCTGCCAACTTC | XhoI |
